# Transesophageal Echocardiography-Guided Thrombectomy of Level IV Renal Cell Carcinoma without Cardiopulmonary Bypass

**DOI:** 10.21470/1678-9741-2018-0216

**Published:** 2019

**Authors:** Petar Zlatanovic, Igor Koncar, Nenad Jakovljevic, Dejan Markovic, Aleksandar Mitrovic, Lazar Davidovic

**Affiliations:** 1 Clinic for Vascular and Endovascular Surgery, Clinical Center of Serbia, Belgrade, Serbia.; 2 Faculty of Medicine, University of Belgrade, Serbia.; 3 Center for Anesthesiology, Clinical Center of Serbia, Belgrade, Serbia.

**Keywords:** Renal Cell Carcinoma, Inferior Vena Cava, Transesophageal Echocardiogram

## Abstract

Advanced renal cell carcinoma accompanied by tumor thrombus in the venous system is present in up to 10% of cases. Extension of tumor thrombus above the diaphragm or into the right atrium represents level IV disease. Level IV tumors are typically treated with sterno-laparotomy approach with or without deep hypothermic circulatory arrest and veno-venous bypass. In this case report, the surgical technique for the resection of advanced RCC were described, with the concomitant use of transesophageal echocardiography for thrombus extraction without the veno-venous or cardiopulmonary bypass.

**Table:** 

Abbreviations, acronyms & symbols	
ARDS	= Acute respiratory distress syndrome
CT	= Computerized tomography
IVC	= Inferior vena cava
LV	= Left ventricular
PA	= Pulmonary arteries
RA	= Right atrium
RCC	= Renal cell carcinoma
TEE	= Transesophageal echocardiography
TTE	= Transthoracic echocardiography

## INTRODUCTION

At the time of diagnosis, renal cell carcinoma (RCC) is accompanied by tumor thrombus in the venous system in up to 10% of cases^[1]^. Resection of RCC with inferior vena cava (IVC) tumor extension poses a challenge with perioperative morbidity and mortality reaching 50%^[2]^. Complete surgical removal remains the only potential hope for a cure. Incomplete resection results in 100% 5-year mortality^[3]^. Extension of tumor thrombus above the diaphragm or into the right atrium (RA) represents level IV disease, according to the Mayo Classification system^[4]^. In this case report, the surgical technique with the concomitant use of transesophageal echocardiography (TEE) for the resection of advanced RCC were described, without the use of venovenous or cardiopulmonary bypass.

## CASE REPORT

**Fig. 1:**
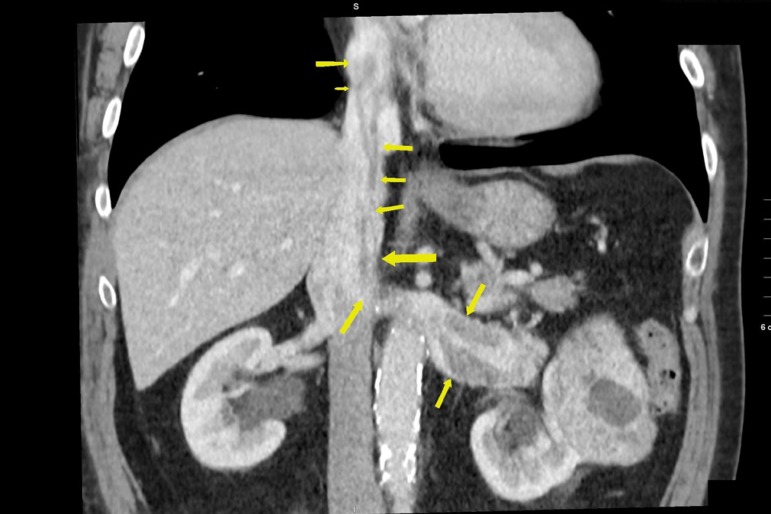
CT scan of male patient with left kidney tumor and thrombus propagation up to the right atrium.

Two patients operated at our clinic in January 2018 were a 55-year-old female and 61-year-old male who were recently diagnosed with RCC, both with stage T4NxM0 (T4 tumor grossly extends into the vena cava above the diaphragm, Nx regional lymph nodes cannot be assessed, and M0 indicates no distal metastasis), who were scheduled for radical right nephrectomy with IVC thrombectomy. All patients were preoperatively evaluated with routine blood analyses, chest X-ray, transthoracic echocardiography (TTE) computerized tomography (CT) and contrast venacavography. Preoperative CT and TTE revealed the tumor extension into the IVC up to the RA for male patient and supradiaphragmatic portion of IVC in female patient (Figure 1).

**Fig. 2:**
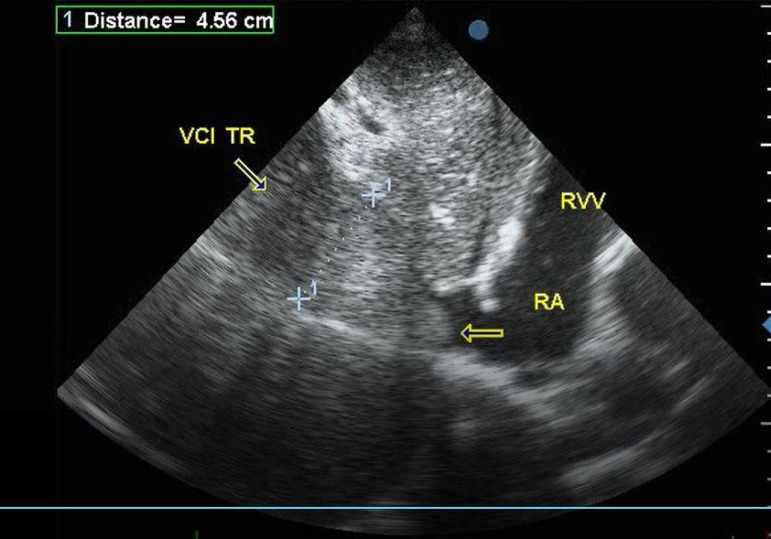
Echocardiogram in female patient with IVC tumor and supradiaphragmatic propagation.

The operation was performed by our team of vascular surgeons. Cardiac surgery team was also assembled assigned to the care of the patient, because the original plan was to do the sterno-laparotomy and partial veno-venous bypass. Following the induction of general anesthesia, internal jugular central line was inserted. In addition, a multiplane TEE probe was placed in the esophagus. Initial TEE examination of the heart confirmed the normal cardiac structures and function without a patent foramen ovale. TEE study was performed with emphasis on the hepatic veins, IVC, right side of the heart, and pulmonary arteries (PAs). The tumor was well visualized in the IVC extending proximal into the RA for male patient and supradiaphragmatic portion of IVC in female patient (Figure 2).

**Fig. 3:**
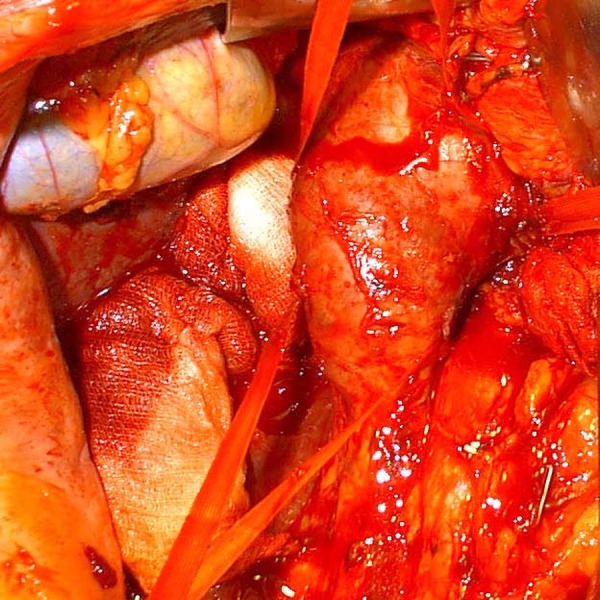
Presentation of inferior vena cava in male patient with left kidney tumor and thrombus propagation up to the right atrium.

**Fig. 4:**
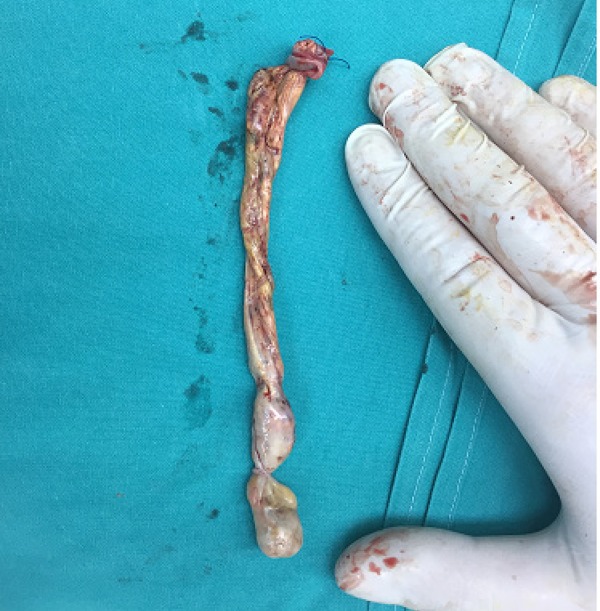
Tumor thrombus from female patient extending to the supradiaphragmatic portion of IVC.

Surgery proceeded with median laparotomy and right subcostal incision for female patient and bilateral subcostal Chevron incision for male patient. Cardio surgical team and cardiopulmonary bypass team were placed on standby. Liver was mobilized, hepatic veins were exposed, and the IVC was isolated (Figure 3). With a Kocher maneuver the infrarenal IVC was exposed. Proximal clamp was placed at the suprahepatic portion of IVC and distally at infrarenal part of IVC. Renal artery and vein were ligated and thereafter infrarenal IVC was clamped and opened at the level of renal vein (right for female and left for male patient). Under the surveillance of TEE, the right atrium thrombus immediately retracted in the supradiaphragmatic part of IVC in male patient and in infradiaphragmatic-suprahepatic portion of IVC in female patient, and, subsequently, thrombectomy was done. After the extraction of the thrombus the nephrectomy was done. On the control TEE there was no remnant thrombus in IVC nor the evidence of distal embolization. The site of thrombectomy on IVC was reconstructed with direct suture in both cases. Due to adhesions between spleen and the left kidney, the spleen was injured in male patient and subsequent splenectomy was done. During the operation the female patient bleed out 2500ml and the male 1500ml. One thousand ml of autologous blood was returned with Cell saver for female patient and 600ml for male patient. The intraoperative course was uneventful and the patient went to the ICU in stable condition. The gross specimen was inspected and found no missing a tissue fragment (Figure 4). Further postoperative TEE revealed no tumor masses in right atrium or retrohepatic part of IVC. Postoperative recovery was uneventful as the first follow-up visit after one month for both patients. Pathohistology two weeks after the operation showed the confirmation of renal cell carcinoma both Fuhrman grade III.

## DISCUSSION

Surgical resection remains treatment mainstay for RCC. The critical factor for successful surgery is mainly management of the IVC, and the important goals are to minimize bleeding and prevent embolism from the thrombus during surgery. Either event often leads to fatal consequences. Over the years, several approaches have been developed to aid in the safe removal of these tumors. The improvement in surgical technique has often been due to the application of surgical principles from different disciplines. Cardiopulmonary bypass, with or without deep hypothermic circulatory arrest, is an example of using cardiac surgery tools in an oncologic operation. However, novel surgical techniques borrowed from liver transplant surgery have allowed for exposure and isolation of the IVC during tumor resection thereby sparing the use of vascular bypass^[5,6]^. The concept of resorting to an entirely intra-abdominal approach without cardiopulmonary or veno-venous bypass is the byproduct of this approach^[2]^. Whereas such techniques avoid the risks of cardiovascular bypass (*i.e*., coagulation system disturbances, longer operating times, systemic inflammatory response), the chance for hemodynamic compromise is increased during vena cava exploration and venous return interruption.

Eighty-five patients were operated because of RCC tumor with extension into the IVC from 2007 to 2017 at the Clinic for Vascular and Endovascular Surgery. Out of those, five patients had the level IV tumor extension of RCC tumor. All of these were operated with the standard use of sterno-laparatomy approach. The clinical perioperative data of the five patients are summarized in Table 1. In all of them partial cavo/veno-venous bypass was used. The duration of operative time, IVC clamping and amount of blood loss was higher in patient treated with standard approch. Two of the patients died intrahospitaly [one due to renal failure and another because of acute respiratory distress syndrome (ARDS)].

**Table 1:** Clinical and perioperative data of all patients with level IV tumor treated at the Clinic for Vascular and Endovascular Surgery.

Parameters	Case 1	Case 2	Case 3	Case 4	Case 5	Case 6	Case 7
Age/Sex	54/M	62/M	60/F	51/M	66/M	61/M	55/F
Clinical Stage	T4N0M0	T4N2M0	T4N0M0	T4N1M0	T4N0M0	T4N0M0	T4N0M0
Laterality of Primary Tumor	Right	Right	Left	Right	Left	Left	Right
Operative Time	6h 21min	5h 11min	5h 42min	4h 57min	6h 02min	3h 19min	3h 33min
Time of IVC Clamp (min)	1h 13min	52	58	48	1h 1min	16	27
Cavo/Veno - Atrial Bypass	Yes	Yes	Yes	Yes	Yes	No	No
Blood Loss (ml)	7500	5700	6050	5200	6850	1600	2500
Intervention Under transesophageal echocardiography Monitoring	None	None	None	None	None	Fogarty Catheter	Fogarty Catheter
30-Day Mortality	Yes	No	No	No	Yes	No	No

With this new concept, RCC with tumor thrombus extending to the supradiaphragmatic IVC can be resected while employing a series of clamps in succession on the IVC that allow for minimal venous return interruption. Once the renal artery is ligated and IVC clamped in infrarenal position and opened, the thrombi are quickly removed with the help of Fogarty catheter or resected to a position in the IVC below the hepatic veins. After the tumor thrombus removal and nephrectomy the liver inflow and outflow is restored. This partially mitigates the hemodynamic disturbance of impaired lower abdominal venous return. Using these techniques, both hemodynamic an ischemic consequences are lessened but still require care from the anesthesiologist in the form of invasive monitors including arterial line, high volume central line, use of vasopressors, and TEE.

Another interesting momment was observed the renal artery ligation and clamping of the infrarenal part of IVC. The thrombus subsequently retracted and allowed its safe removal. This could be explained that the most thrombus are not adherent to IVC wall, and also by the fact that the inflow to the RA is reduced allowing thrombus not to bee pushed by "full blood flow". This maneuver "simplify" operation when the proximal extent of tumor is near hepatic veins or right atrium^[4]^.

TEE has major effects on the decision-making process in surgical and anesthetic management. TEE can also demonstrate the tumor mobility, whether it is fragile and/or adherent to the IVC. A fragile tumor has a higher risk of pulmonary embolism, and adherence of the tumor to the IVC wall may result in an incomplete resection of the tumor or vascular injury when using an occlusion balloon catheter. TEE imaging can determine whether it will be possible to bring the thrombus down with the balloon or not, and also provide information about the precise location and size of the balloon. Because TEE visualized a mobile and oscillating thrombus, we decided to remove it by using an occlusion balloon catheter. We removed the thrombus successfully by this simple technique with minimum hepatic mobilization for the thrombectomy. It should be also monitored by the operators directly on the surgical field. Intraoperative real-time TEE monitoring can be provided by anesthesiologists without interruption of the surgical procedure, and we regard this as one of the most important advantages of TEE monitoring. Most importantly, the real-time TEE monitoring allowed us to perform this intervention safely. Fortunately, no evidence of pulmonary embolism was found in our two cases. Nevertheless real-time TEE can provide rapid recognition of massive pulmonary embolism. TEE can reveal the sudden disappearance of the tumor head, presence of the thrombus in the pulmonary artery, or any sign of right heart dysfunction. Intraoperative TEE is also useful in anesthetic management to evaluate the left ventricular volume (LV) and function. Massive bleeding and decreased venous return due to the IVC clamp can be immediately treated with volume loading according to the LV volume assessed by TEE.

## CONCLUSION

Our preliminary experience suggest that the advantages of intraoperative TEE, including verification of the level of IVC involvement, confirmation of complete removal of the IVC thrombus, and intervention using catheters to assist in thrombectomy. This would help with earlier detection and management of complications, which will probably contribute to a decrease in the morbidity and mortality associated with the management of those high-risk patients.

**Table:** 

Authors' roles & responsibilities	
PZ	Study conception and design; acquisition of data; analysis and interpretation of data; drafting of manuscript; final approval of the version to be published
IK	Study conception and design; analysis and interpretation of data; drafting of manuscript, critical revision; final approval of the version to be published
NJ	Analysis and interpretation of data; critical revision; final approval of the version to be published
DM	Acquisition of data; drafting of manuscript; final approval of the version to be published
AM	Acquisition of data; drafting of manuscript; final approval of the version to be published
LD	Study conception and design; analysis and interpretation of data; drafting of manuscript; critical revision; final approval of the version to be published
